# Identification of a novel β-adrenergic octopamine receptor-like gene (*βAOR*-like) and increased ATP-binding cassette B10 (*ABCB10*) expression in a *Rhipicephalus microplus* cell line derived from acaricide-resistant ticks

**DOI:** 10.1186/s13071-016-1708-x

**Published:** 2016-08-02

**Authors:** H. H. Caline Koh-Tan, Erin Strachan, Katherine Cooper, Lesley Bell-Sakyi, Nicholas N. Jonsson

**Affiliations:** 1 Institute of Biodiversity, Animal Health and Comparative Medicine, University of Glasgow, McCall Building, Bearsden Road, Glasgow, G61 1QH UK; 2 School of Veterinary Medicine, University of Glasgow, Glasgow, G61 1QH UK; 3 The Pirbright Institute, Ash Road, Pirbright, Woking, Surrey, GU24 0NF UK

**Keywords:** Acaricide resistance, Amitraz, Synthetic pyrethroid, Ivermectin, Dieldrin, Tick cell line

## Abstract

**Background:**

The cattle tick *Rhipicephalus* (*Boophilus*) *microplus* is an economically important parasite of livestock. Effective control of ticks using acaricides is threatened by the emergence of resistance to many existing compounds. Several continuous *R. microplus* cell lines have been established and provide an under-utilised resource for studies into acaricide targets and potential genetic mutations associated with resistance. As a first step to genetic studies using these resources, this study aimed to determine the presence or absence of two genes and their transcripts that have been linked with acaricide function in cattle ticks: β-adrenergic octopamine receptor (*βAOR*, associated with amitraz resistance) and ATP-binding cassette B10 (*ABCB10*, associated with macrocyclic lactone resistance) in six *R. microplus* cell lines, five other *Rhipicephalus* spp. cell lines and three cell lines representing other tick genera (*Amblyomma variegatum*, *Ixodes ricinus* and *Hyalomma anatolicum*).

**Methods:**

End-point polymerase chain reaction (PCR) was used for detection of the *βAOR* gene and transcripts in DNA and RNA extracted from the tick cell lines, followed by capillary sequencing of amplicons. Quantitative real-time PCR (qPCR) was performed to determine the levels of expression of *ABCB10*.

**Results:**

*βAOR* gene expression was detected in all *Rhipicephalus* spp. cell lines. We observed a second amplicon of approximately 220 bp for the *βAOR* gene in the *R. microplus* cell line BME/CTVM6, derived from acaricide-resistant ticks. Sequencing of this transcript variant identified a 36 bp insertion in the *βAOR* gene, leading to a 12-amino acid insertion (LLKTLALVTIIS) in the first transmembrane domain of the protein. In addition, nine synonymous SNPs were also discovered in *R. appendiculatus*, *R. evertsi* and *R. sanguineus* cell lines. Some of these SNPs appear to be unique to each species, providing potential tools for differentiating the tick species. The BME/CTVM6 cell line had significantly higher *ABCB10* (*P* = 0.002) expression than the other *R. microplus* cell lines.

**Conclusions:**

The present study has identified a new *βAOR* gene and demonstrated a higher *ABCB10* expression level in the BME/CTVM6 cell line, indicating that tick cell lines provide a useful experimental tool for acaricide resistance studies and further elucidation of tick genetics.

**Electronic supplementary material:**

The online version of this article (doi:10.1186/s13071-016-1708-x) contains supplementary material, which is available to authorized users.

## Background

*Rhipicephalus* (*Boophilus*) *microplus*, also known as the cattle tick or southern cattle tick, is a parasite of major global economic importance. Sales-Junior et al. [1] estimated that more than 75 % of the cattle population worldwide was infested with *R. microplus*, making it one of the most prevalent of bovine parasites. *Rhipicephalus microplus* causes significant economic losses; direct losses include damage to the hide from tick attachment, anaemia and in severe cases death [2]. More importantly, cattle ticks act as vectors of the protozoan and bacterial pathogens *Babesia bigemina*, *Babesia bovis* and *Anaplasma marginale*, all of which cause production losses and can be fatal [3]. *Rhipicephalus appendiculatus* is an important tick of cattle in eastern Africa, where it transmits *Theileria parva* [4]. *Rhipicephalus sanguineus* is a globally widespread tick that is commonly found on dogs [5]. *Amblyomma variegatum* infests cattle throughout sub-Saharan Africa and transmits *Ehrlichia ruminantium*, the causal agent of heartwater [6]. *Ixodes ricinus* is the most widely distributed tick species in northern, western and central Europe and is of human health significance as the vector of Lyme disease (*Borrelia burgdorferi*) as well as being an important vector of several diseases of cattle and sheep [4, 7]. *Hyalomma anatolicum* infests cattle in North Africa, the Middle East and Asia and transmits *Theileria annulata*, of economic significance in the region [4].

The short, one-host life-cycle of *R. microplus* acts together with high fecundity to result in rapid selection for development of acaricide resistance. There are several mechanisms by which acaricide resistance has evolved; those most commonly noted are target site modification and metabolic resistance [8–10]. Target site modification has been most clearly demonstrated in the *para* sodium channel, the target of synthetic pyrethroids (SP) [11, 12]. Metabolic resistance occurs as a result of modified ability to detoxify and/or sequester pesticides, mediated by families of enzymes known for detoxification of xenobiotics, such as the cytochrome P450s, esterases, glutathione S-transferases and ATP-binding cassette (ABC) transporters [10]. Penetration resistance is a little-studied mechanism which might also occur in ticks [9].

Octopamine receptors have been shown from studies in insects to be the target for formamidines such as amitraz, leading to the hypothesis that mutations in octopamine receptors would confer resistance to formamidines [13]. There are three main types of octopamine receptors in insects: α-adrenergic-like, β-adrenergic-like and tyramine/octopamine (tyraminergic) [14]. Our previous work reported an association between polymorphism in the β-adrenergic octopamine receptor (*βAOR*) and resistance to amitraz in *R. microplus* ticks in Australia [15]. A non-synonymous A → T at position 181 resulted in substitution of isoleucine to phenylalanine (I61F) in the first trans-membrane domain. It is therefore expected that any resistance conferred by mutation in βAOR would be restricted to compounds that would bind to this receptor type.

ATP-binding cassette (ABC) transporters are a superfamily of transmembrane proteins, consisting of up to 8 subfamilies [16] found in all organisms, including *R. microplus* [17]. They transport substrates across cell membranes as a monomeric or dimeric channel, often against concentration gradients [16]. An association between the ABC transporter and ivermectin (macrocyclic lactone) resistance amongst cattle ticks was first reported by Pohl et al. [17]. These authors detected a 3–6-fold increase in gene expression of *R. microplus* ABC subtype B10 (*RmABCB10*) in two different ivermectin-resistant strains. The importance of increased *ABCB10* expression through metabolic processes was subsequently demonstrated in an ivermectin resistance-induced *R. microplus* cell line [18] but it is uncertain if *ABCB10* plays a role in resistance to other classes of acaricides.

Over 40 continuous tick cell lines derived from species of worldwide economic importance are maintained in the Tick Cell Biobank at The Pirbright Institute [19]. The establishment of tick cell lines is slow and challenging, and the cultures are composed of cells obtained from hundreds or thousands of embryos or multiple developing nymphs or adults. Further, the cultures are composed of multiple cell types and the cells can be of variable ploidy [20], with proportions of the different cell populations in a cell line varying over time. Therefore, although a small number of acaricide resistance selection studies have been successfully conducted using continuous tick cell lines [21, 22], because they are non-clonal and phenotypically and genotypically heterogeneous it cannot be assumed that any continuous tick cell line is a reliable and consistent model for examining acaricide resistance.

The purpose of the present study was to assess the suitability of six *R. microplus* continuous cell lines for future genomic and transcriptomic studies on resistance to acaricides. In this study we wished to confirm that two genes that have been strongly associated with resistance (one that operates via target site modification and one that operates via metabolic resistance mechanisms) are expressed by tick cells in vitro, to identify new variants of these genes if any were present, and to determine whether there is variation in their expression level. These two genes were selected for this study because (i) amitraz and macrocyclic lactones (including ivermectin) are globally the most widely used acaricides at present, (ii) we have well-established protocols for working with these genes, and (iii) they represent two of the main mechanisms of resistance-detoxification (*ABCB10*) and target-site insensitivity (*βAOR*). The first objective was to partially sequence the *βAOR* gene for single nucleotide polymorphisms (SNPs) that were reported previously by Corley et al. [15]. The second objective was to determine the expression level of *ABCB10* in the *Rhipicephalus* spp., in particular the *R. microplus*, cell lines. The study design did not allow a robust determination of the relationship between genotype or expression level and the resistance status of the population of ticks from which the continuous cell lines were developed.

## Methods

### Cell lines

Fourteen tick cell lines, derived from *R. microplus*, three other *Rhipicephalus* spp., *A. variegatum*, *H. anatolicum* and *I. ricinus*, were provided by the Tick Cell Biobank at The Pirbright Institute. The cell lines, passage level(s) tested, species and instar of origin, geographic origin and acaricide resistance status of the parent ticks (if known) are listed in Table 1. All tick cell lines were maintained in 2 ml complete culture medium supplemented with foetal bovine serum in flat-sided culture tubes (Nunc) [23]. Prior to nucleic acid extraction, the tubes were completely filled with culture medium and transferred to the University of Glasgow at room temperature. On arrival, the entire contents of each tube of cells was centrifuged at 300 *rcf* for 5 min, most of the supernatant medium was discarded, leaving only 3 ml of medium to re-suspend the cells. The cells were then divided into 3 aliquots in 1.5 ml microtubes and centrifuged at 900 *rcf* for 5 min. The supernatants were then discarded and the cell pellets frozen at −80 °C for nucleic acid extraction.

**Table 1:** Tick cell lines used in the study

Species	Cell line	Passage level tested	Instar of origin	Year initiated	Geographical origin (strain)	Reference	Acaricide resistance status of parent ticks if known
*Rhipicephalus* (*Boophilus*) *microplus*	BmVIII-SCC	41	Embryo	1979	Mexico	[32]	Susceptible
	BME/CTVM2	140	Embryo	1983	Costa Rica (Paquera)	[33]	Susceptible
	BME/CTVM5	67; 8	Embryo	1983	Colombia (Paso Ancho)	[20]	Resistant to organophosphates, organochlorines, Amitraz
	BME/CTVM6	211/233; 32; 221; 243	Embryo	1983	Colombia (Paso Ancho)	[33]	Resistant to organophosphates, organochlorines, Amitraz
	BME/CTVM23	54	Embryo	2005	Mozambique (Mozambique)	[34]	Not known
	BME/CTVM30	17	Embryo	2005	Mozambique (Mozambique)	[34, 35]	Not known
*Rhipicephalus appendiculatus*	RAN/CTVM3	66	Developing adult	1978	Kenya (Muguga)	[36]	Not known
	RA243	344	Developing adult	1971	East Africa	[37]	Not known
*Rhipicephalus evertsi*	REN/CTVM32	16	Developing adult	2010	South Africa	[23]	Not known
*Rhipicephalus sanguineus*	RML-RSE	83	Embryo	1980s	United States of America	[38, 39]	Not known
	RSE/PILS35	4	Embryo	2012	France	This study	Not known
*Amblyomma variegatum*	AVL/CTVM13	127	Developing nymph	1989	Southern Africa	[40]	Not known
*Hyalomma anatolicum*	HAE/CTVM9	195	Embryo	1986	India (Ludhiana)	[41]	Not known
*Ixodes ricinus*	IRE/CTVM19	222	Embryo	1999	United Kingdom	[20]	Not known

All cell lines were grown in culture media and at incubation temperatures as described by Bell-Sakyi et al. [23] except RSE/PILS35, which was established in L-15 medium at 28 °C from eggs laid by a single incompletely-engorged female *R. sanguineus* tick kindly provided by Dr Cristina Socolovschi and Dr Oleg Mediannikov, URMITE, Marseille, France

### Extraction of nucleic acids

Total RNA was extracted from cell pellets using the Qiagen miRNeasy Mini Kit (#217004) with on-column DNase digestion following the manufacturer’s protocol for extraction from cells. Genomic DNA (gDNA) was extracted from cell pellets using the Qiagen QiaAMP Mini Kit (#51304) following the manufacturer’s protocol. The RNA and DNA concentrations were measured spectrophotometrically using a Nanodrop ND-1000 and quality was confirmed at absorbance 260/280 nm to be ≥ 1.95.

### Reverse transcription

First strand complementary DNA (cDNA) was synthesised using 800 ng total RNA with TaqMan Reverse Transcription Reagents (Life Technologies #N8080234) in a 20 μl reaction volume according to the manufacturer’s instructions. This was performed in a Life Technologies SimpliAmp™ Thermal Cycler using the recommended cycling conditions. The cDNA was then diluted with 30 μl of RNase-free water, before any subsequent experiments.

### Polymerase chain reaction (PCR)

Each reaction was set up using a HotStar Taq Plus DNA Polymerase kit (Qiagen #203603) in a 20 μl volume containing final concentrations of 1× buffer (with 1.5 mM MgCl_2_), dNTPs (0.2 mM), forward primer (0.5 μM), reverse primer (0.5 μM), Taq polymerase (5 U/μl), RNase-free H_2_O and 2 μl of cDNA or 20 ng of gDNA. The primers and PCR conditions used are shown in Table 2. A non-template control (NTC) was included in every PCR. End-point PCR for *ABCB10* and the housekeeping gene *β-actin* (*ActB*) using cDNA of all cell lines was done to check for presence of transcript before proceeding to quantitative PCR (qPCR). Thermal cycling conditions were initial activation at 95 °C for 5 min; 35 cycles of 95 °C for 1 min, annealing temperature (Table 2) for 1 min at 72 °C; and final extension at 60 °C for 30 min.

**Table 2:** Primers and PCR conditions

Oligo name	Sequence (5′-3′)	AT	Amplicon	Accession No.	Positions	Reference
*βAOR*-For	GAAATCTGACGGACGAGGAA	61 °C (Rm)	183 bp	JN974909	95–277	[15]
*βAOR*-Rev	GCGACACGATGAAGTAGTTG	58 °C (Rhipi)				
*ABCB10*-For	GCCGCAGTTGTCACTTGTTGGTTTG	61 °C	96 bp	JN098446	887–982	[17]
*ABCB10*-Rev	ACGTCCGCTGCCACTTGCCTC					
*ActBm1*-For	GAGGAAGTACTCCGTCTGGATCGG	61 °C	203 bp	AY255624	1095–1297	[42]
*ActBm1*-Rev	CCGTAGGGTGGCGTTGCCGG					

*Abbreviations*: *AT* annealing temperature, *bp* base pair, *Rm Rhipicephalus microplus*, *Rhipi* other *Rhipicephalus* species

### Sequencing of *βAOR*

All detected amplicons from gDNA and cDNA, with the exception of gDNA from BME/CTVM5, were cleaned for sequencing using Agencourt AMPure XP (Beckman Coulter #A63880) according to the manufacturer’s instructions and reconstituted in 40 μl of RNase-free water. For cell line BME/CTVM5, only the amplicon from cDNA was sequenced because the other two genomic fragments that were amplified were not transcribed. Cleaned amplicons were checked by 1.5 % agarose gel electrophoresis before sequencing. Sequencing reactions were set up using HPLC-purified primers and a BigDye® Terminator v3.1 Cycle Sequencing Kit (Life Technologies #4337455) in a 20 μl volume containing a final concentration of 3.2 pmol of forward or reverse primer, 0.5 μl of BigDye reaction mix, 3.5 μl of 5× sequencing buffer and 10 μl of cleaned amplicon.

Sequencing reactions were cleaned using an Agencourt CleanSEQ Dye Terminator Removal Kit (#A29151) according to the manufacturer’s instructions, and trace signal was detected through capillary electrophoresis on a 3130XL Genetic Analyzer. CLC Genomics Workbench 7.5.2 was used to manually check the trace results, generate alignments and the amino acid sequence used for domain prediction and 2-dimensional representation of protein structure.

### Real-time quantitative PCR (qPCR)

Real-time quantitative measurements of *ABCB10* and *ActB* expression were determined in a 10 μl reaction volume containing 1X Brilliant III Ultra-Fast SYBR Green Q-PCR Master Mix (Agilent Technologies #600882), 0.03 μM of ROX reference dye, 0.5 μM forward and reverse primers (Table 2) and 2 μl of cDNA. A non-template control (NTC) was included in every run. Each sample was set up in technical duplicates. The qPCR was performed using the real-time system Stratagene MX3000p and cycling conditions were initial denaturation at 95 °C for 5 min; 35 cycles of 95 °C for 1 min, 60 °C for 1 min and 72 °C for 1 min; final extension at 60 °C for 30 min; and finally a melting curve for confirmation of single amplicon. The cycle threshold (Ct) values were exported for analysis in Excel. The experiment was replicated twice with all nine cell lines investigated and twice more with only *R. microplus* cell lines.

### Statistical analysis

Expression of the *ABCB10* gene was normalised to *ActB* expression and the mean ratio for technical duplicates calculated based on the Pfaffl method [24], using BmVIII-SCC as the calibrator cell line. Differences between the cell lines were tested using one-way analysis of variance (ANOVA) with Tukey’s *post-hoc* test on Minitab v17.

## Results

### Detection of the *β*AOR gene and transcripts in tick cell lines

**Fig. 1:**
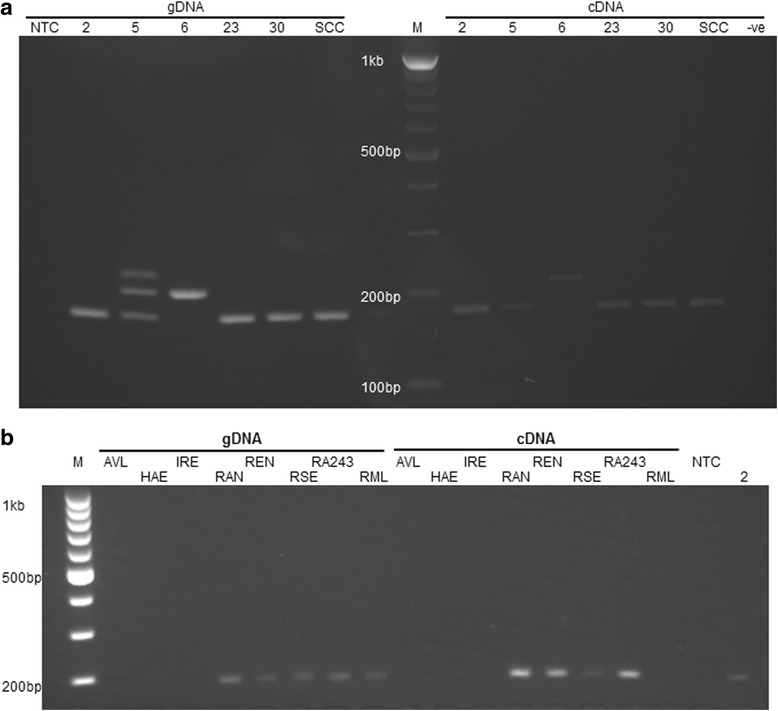
*βAOR* PCR in *Rhipicephalus* cell lines. **a** Detection of *βAOR* in genomic DNA (gDNA) and complementary DNA (cDNA) of *Rhipicephalus microplus* cell lines. The expected amplicon of 183 bp was detected in gDNA and cDNA of cell lines BME/CTVM2 (2), BME/CTVM5 (5), BME/CTVM23 (23), BME/CTVM30 (30) and BmVIII-SCC (SCC). BME/CTVM6 (6) gave an alternative amplicon of ~220 bp from both gDNA and cDNA. Additional amplicons of ~245 bp and ~220 bp were also detected in gDNA of BME/CTVM5. **b** Detection of *βAOR* in gDNA and cDNA of other tick cell lines. The expected amplicon was detected in gDNA and cDNA of cell lines RAN/CTVM3 (RAN), REN/CTVM32 (REN), RSE/PILS35 (RSE) and RA243, and only in gDNA of RML-RSE (RML). No *βAOR* gene or transcript was detected in cell lines AVL/CTVM13 (AVL), HAE/CTVM9 (HAE) or IRE/CTVM19 (IRE). *Abbreviations*: M, Marker; NTC, Non-template control; −ve, reverse transcription negative control

The *βAOR* gene and transcripts from the six *R. microplus* cell lines were detected by PCR in gDNA and cDNA respectively (Fig. 1a). An amplicon of the expected size, 183 bp, was detected in gDNA and cDNA of BME/CTVM2, BME/CTVM5 (passage 67), BME/CTVM23, BME/CTVM30 and BmVIII-SCC. An alternative amplicon of ~220 bp was detected in gDNA and cDNA of BME/CTVM6 (mixture of cells from two sublines at passages 211 and 233, separate since passage 148). There were also two additional amplicons of ~245 bp and ~220 bp in gDNA of BME/CTVM5 (passage 67); these were not detected in cDNA from this cell line (Fig. 1a). To determine whether or not the alternative/additional amplicons detected in BME/CTVM5 and BME/CTVM6 cells were consistently present in these cell lines at different passage levels, the earliest available passages of BME/CTVM5 (passage 8) and BME/CTVM6 (passage 32) and from the two high-passage sublines of BME/CTVM6 (passages 221 and 243 extracted separately) were tested. In all cases the respective amplicons detected in gDNA from the first high passages tested were also present in the respective low passages and the two sublines of BME/CTVM6 (Additional file 1: Figure S1).

Amplicons of the expected 183 bp size were detected in gDNA and cDNA of RAN/CTVM3, RA243, REN/CTVM32 and RSE/PILS35, but only in gDNA of RML-RSE (Fig. 1b). The *βAOR* gene and transcripts were not detected in cell lines AVL/CTVM13, HAE/CTVM9 and IRE/CTVM19 (Fig. 1b) using the primers in Table 2 in two attempts. Consequently, these three cell lines were excluded from further investigation.

### *βAOR* gene variations

**Fig. 2:**
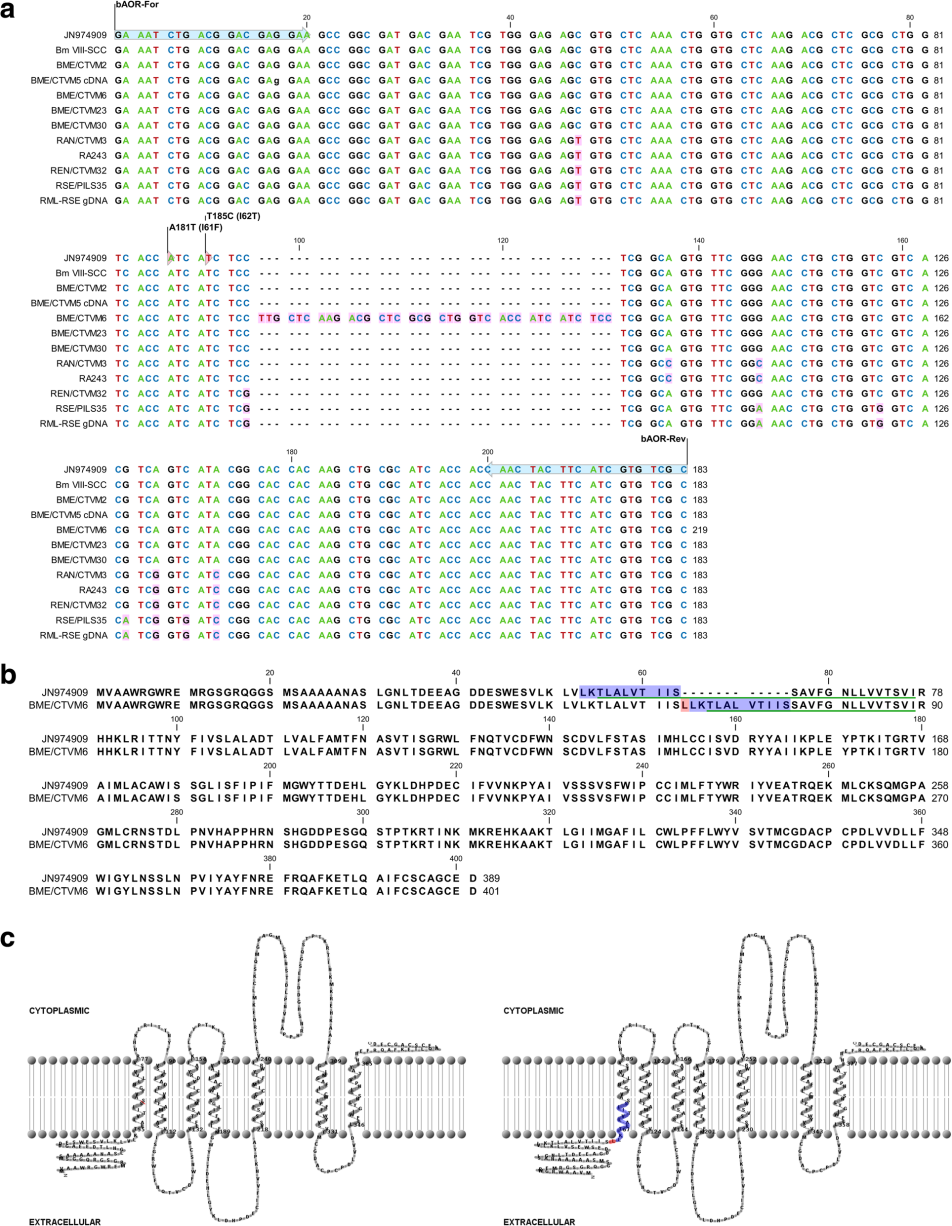
Alignment of *Rhipicehalus βAOR* sequences and predicted structure of the βAOR protein. **a** Alignment of *βAOR* genomic sequences in *Rhipicephalus* spp. cell lines with published *R. microplus* strain NRFS (JN974909). Variations are highlighted in pink. **b** Alignment of *βAOR* amino acid sequences of published *R. microplus* strain NRFS (JN974909) and BME/CTVM6. The 12-aa insertion consists of an additional L (highlighted in *red*), followed by what appears to be duplication of LKTLALVTIIS (highlighted in *blue*). Green lines indicate the first transmembrane domain. **c** 2-dimensional representation of the *R. microplus* strain NRFS βAOR protein (*left diagram*) and the consequence of the 12-aa insertion (highlighted in *red* and *blue*) in the BME/CTVM6 βAOR protein (*right diagram*). The *red line* in the left diagram indicates the position of insertion. Membrane-spanning domains were predicted by the TMHMM Server at the Center for Biological Sequence Analysis, Technical University of Denmark, DTU (http://www.cbs.dtu.dk/services/TMHMM/) and 2 dimensional representation by TMRPres2D [31]

Sequencing and comparison with the published sequence (GenBank JN974909) of the *βAOR* gene of *R. microplus* (strain NRFS) revealed that the transcript variant in BME/CTVM6 and the 220 bp gene variant in BME/CTVM5 contained a 36 bp insertion at position 190 from the start codon (Fig. 2a), not present in any of the other tick cell lines. This resulted in a 12-aa insertion LLKTLALVTIIS (Fig. 2b), which appeared to be an additional leucine (L) followed by a duplicated LKTLALVTIIS. Using the TMHMM prediction and TMRPres2D diagram representation, this insertion was predicted to occur within the first transmembrane domain leaving an extracellular domain comprising 66-aa instead of 54-aa (Fig. 2c). A discontiguous megablast search did not find any match for this insertion in any other species.

Attempts to sequence the additional 245 bp amplicon from BME/CTVM5 were unsuccessful despite successful gel extraction of the product (Additional file 2: Figure S2). There were two regions of ambiguity within the amplicon between regions of homology of the sequences, suggesting that there was more than one binding site for the primers. PCR amplification of the purified 245 bp amplicon produced the same three amplicons seen in Fig. 1a and Additional file 1: Figure S1. This confirms that there was more than one binding site for the primers.

Nine synonymous SNPs were also discovered in *R. appendiculatus*, *R. evertsi* and *R. sanguineus* cell lines (Table 3). Three SNPs (C141T, A225G and A231C) were present in all five *R. appendiculatus*, *R. evertsi* and *R. sanguineus* cell lines, while C189G was present only in the *R. evertsi* and *R. sanguineus* lines. A195C and G204C were unique to *R. appendiculatus* cell lines while G204A, C216G, G222A and C228G were unique to *R. sanguineus* cell lines.

**Table 3:** Variations in *Rhipicephalus* bAOR gene

Position	Allele	Consequence	Species – Strain/Cell line	Reference
181	A / T	I / F	*R. microplus* – Ultimo strain	[15]
185	T / C	I / T	*R. microplus* – southeast Queensland, Australia	[15]
141	C / T	synonymous	*R. appendiculatus* – RAN/CTVM3, RA243 *R. evertsi* – REN/CTVM32 *R. sanguineus* – RML-RSE, RSE/PILS35	
189	C / G	synonymous	*R. evertsi* – REN/CTVM32 *R. sanguineus* – RML-RSE, RSE/PILS35	
190	36 bp insertion	12 amino acid insertion (LLKTLALVTIIS)	*R. microplus* – BME/CTVM6	
195	A / C	synonymous	*R. appendiculatus* – RAN/CTVM3, RA243	
204	G / C / A	synonymous	*R. appendiculatus* – RAN/CTVM3, RA243 *R. sanguineus* – RML-RSE, RSE/PILS35	
216	C / G	synonymous	*R. sanguineus* – RML-RSE, RSE/PILS35	
222	G / A	synonymous	*R. sanguineus* – RML-RSE, RSE/PILS35	
225	A / G	synonymous	*R. appendiculatus* – RAN/CTVM3, RA243 *R. evertsi* – REN/CTVM32 *R. sanguineus* – RML-RSE, RSE/PILS35	
228	C / G	synonymous	*R. sanguineus* – RML-RSE, RSE/PILS35	
231	A / C	synonymous	*R. appendiculatus* – RAN/CTVM3, RA243 *R. evertsi* – REN/CTVM32 *R. sanguineus* – RML-RSE, RSE/PILS35	

These sequences have been submitted to GenBank under the following accession numbers: KU836738 (BmVIII-SCC), KU836739 (BME/CTVM2), KU836740 (BME/CTVM5), KU836741 (BME/CTVM6), KU836742 (BME/CTVM23), KU836743 (BME/CTVM30), KU836744 (RAN/CTVM3), KU836745 (RA243), KU836746 (REN/CTVM32), KU836747 (RML-RSE) and KU836748 (RSE/PILS35).

### Expression of *ABCB10*

**Fig. 3:**
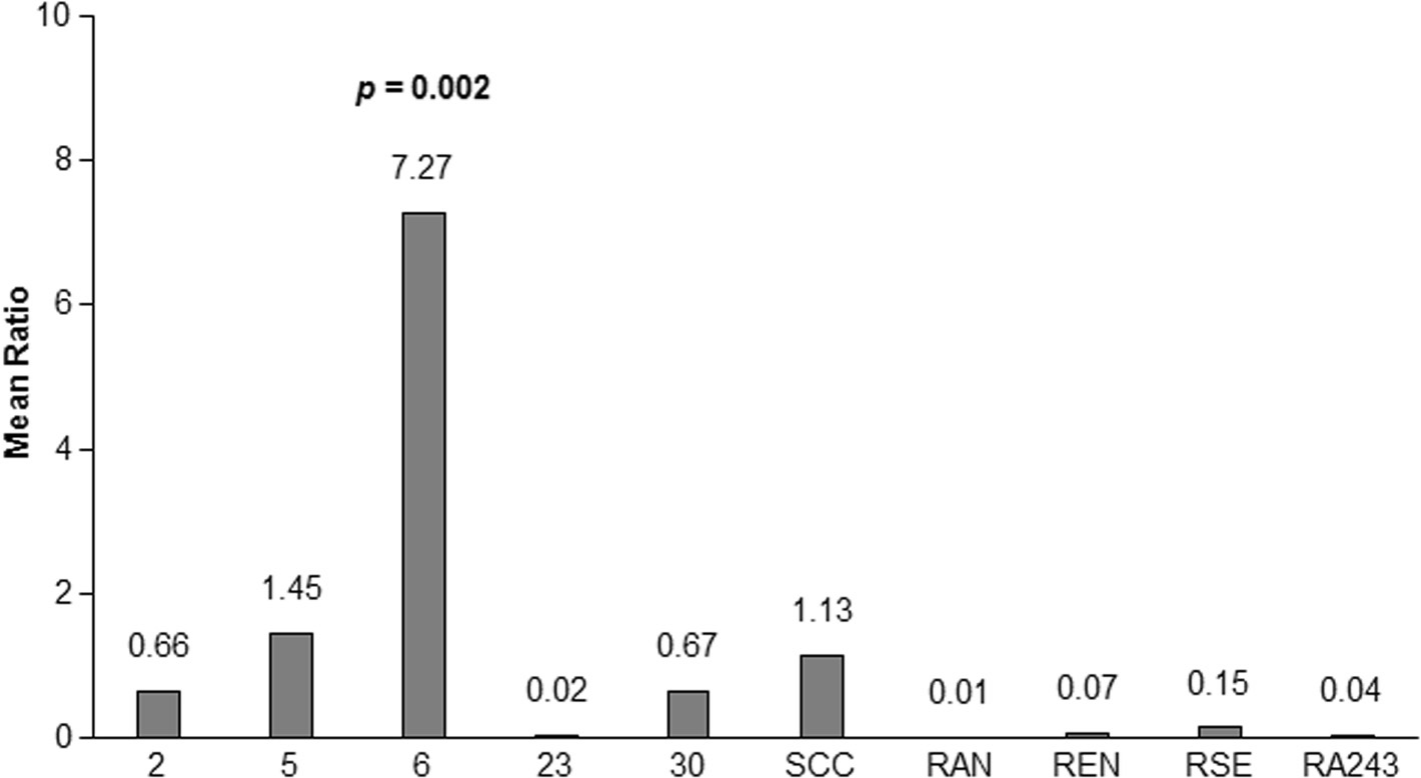
*ABCB10* expression levels in *Rhipicephalus* spp. cell lines. *ABCB10* expression in *Rhipicephalus* spp. cell lines BME/CTVM2 (2), BME/CTVM5 (5), BME/CTVM6 (6), BME/CTVM23 (23), BME/CTVM30 (30), RAN/CTVM3 (RAN), REN/CTVM32 (REN), RSE/PILS35 (RSE) and RA243 using BmVIII-SCC (SCC) as the calibrator cell line. Expression levels are shown numerically above each bar in the graph. Expression of *ABCB10* was significantly higher in BME/CTVM6 (*P* = 0.002) compared to all other cell lines. There was no significant difference in *ABCB10* expression level between all other cell lines. Gene expression is represented as mean ratio of *ABCB10* to *ActB*, calculated according to the Pfaffl method [24], compared using one-way analysis of variance (ANOVA) with Tukey’s *post-hoc* test

After normalisation to *ActB*, expression of the *ABCB10* gene in BME/CTVM6 was significantly higher (ANOVA *F*_(9,10)_ = 8.1, *P* = 0.002), by approximately seven-fold, compared to expression in all the other cell lines (Fig. 3). There was no significant difference in the *ABCB10* expression level between any of the other cell lines.

## Discussion

Acaricide resistance in *R. microplus* has been associated with mutations in several genes including the *βAOR* gene [15]. We demonstrated expression of this gene in six tick cell lines derived from four strains of *R. microplus* with different acaricide resistance status. Corley et al. [15] identified two non-synonymous SNPs for two highly conserved adjacent isoleucine residues of the first transmembrane domain of the β-adrenergic-like octopamine receptor (βAOR) in *R. microplus*. The first identified SNP resulting in an amino acid substitution of I61F was found in the Ultimo tick strain from Australia, which was resistant to amitraz, synthetic pyrethroids and organophosphates. Our study used the same primers and detected the *βAOR* gene and transcripts in all the *R. microplus* cell lines that retain the highly conserved isoleucine at amino acids 61 and 62. However, we discovered an alternative *βAOR* gene and transcript with a 36 bp insertion in the cell line BME/CTVM6, derived from *R. microplus* (Paso Ancho strain) resistant to multiple acaricides. This insertion introduced 12 aa into the first transmembrane domain of the seven-transmembrane protein structure, resulting in a longer extracellular domain. The βAOR protein is the predicted target for formamidines [25]; hence, we hypothesise that the longer extracellular domain could lead to a pore site that is less accessible to formamidines and might confer resistance to them.

The BME/CTVM5 cell line, derived from the same *R. microplus* strain as BME/CTVM6, has potentially three different *βAOR* genes, only one of which was transcribed. The transcribed gene is completely homologous to the published *βAOR* gene JN974909 [25]. One of the other two gene variants appears to give the same fragment size as that of BME/CTVM6 but was not transcribed. Sequencing of this 220 bp amplicon yielded the same sequence as the alternative gene and transcript detected in BME/CTVM6. Multiple attempts to sequence the 245 bp amplicon that was detected only in BME/CTVM5 were unsuccessful, often producing very noisy signals. This suggests multiple primer binding sites within the 245 bp fragment. A repeat PCR performed on the gel-extracted and cleaned 245 bp PCR amplicon from the initial PCR produced the same three amplicons, confirming the existence of multiple binding sites for the primers, most likely due to an evolutionary process of gene duplication. It is notable that despite BME/CTVM5 cells having potentially three variants of the *βAOR* gene, present at both low and high passage levels, only one seems to have been transcribed. As the two cell lines were each derived from different pooled egg batches laid by over 50 female ticks, a possible explanation for this phenomenon is that BME/CTVM5 and BME/CTVM6 were derived from a population of ticks in which resistance was developing but in which the resistance-conferring allelic variants were not fixed at 100 % in the population. The establishment of the two cell lines would likely have been subject to normal stochastic effects on founder populations and subsequent genetic drift, leading to fixation of the initially more frequent allele over time after establishment of the culture. If this is the case, it highlights the potential value of the heterogeneous cell culture model for studies on the evolution of acaricide resistance, and draws attention to aspects of the system that must be taken into consideration in the design of experiments. We hypothesise that the *βAOR* gene was first duplicated in the base population. Gene duplication or multiple gene copy number has been linked to evolutionary adaptation in the parasitic trematode *Fasciola hepatica* [26], the mosquito *Anopheles gambiae* [27] and *R. microplus* [28, 29].

Pohl et al. [18] reported an association between increased expression of ABC transporter genes, *ABCB10* in particular, and ivermectin resistance. BME/CTVM6 demonstrated approximately seven-fold higher *ABCB10* expression when compared to BmVIII-SCC, a cell line derived from acaricide-susceptible ticks (Patricia Holman, personal communication). The importance of *ABCB10* expression level in ivermectin resistance was demonstrated in a *R. microplus* [18] cell line while in an *I. scapularis* cell line, another ABC transporter gene, *ABCB8*, was upregulated [30]. BME/CTVM5 demonstrated a trend towards higher *ABCB10* expression compared to BmVIII-SCC. In order to verify if these increased gene expression levels translate to protein expression levels, further work will be necessary.

It is interesting that the BME/CTVM6 cell line derived from an acaricide-resistant strain has both a different *βAOR* gene and a higher level of *ABCB10* expression compared to the other *R. microplus* cell lines, whereas the BME/CTVM5 cell line, derived from the same strain, did not show these characteristics. It is also notable that both BME/CTVM5 and BME/CTVM6 were established over 30 years ago, prior to the recent heavy and widespread use of macrocyclic lactone products and the documented emergence of ivermectin resistance. It is possible that *ABCB10* played a role in resistance to acaricides other than ivermectins, but it is unlikely that any mutation in *βAOR* would lead to resistance to macrocyclic lactones, as the macrocyclic lactones are not known to bind to *βAOR*. The fact that the same tick strain has returned quite different results in terms of the expression of a detoxifying gene and the genotype of a target gene clearly demonstrates the importance of initial stochastic effects at the time of the establishment of the cell culture.

Nine synonymous SNPs were found in *R. appendiculatus*, *R. evertsi* and *R. sanguineus* cell lines. Some of these SNPs appear to be unique to individual species. These SNPs provide a potential tool for identifying the tick species; however, they need to be verified with *R. appendiculatus*, *R. evertsi* and *R. sanguineus* ticks from different regions of the world and with known acaricide resistance status.

Failure to detect the *βAOR* gene in gDNA extracted from the *Amblyomma*, *Hyalomma* and *Ixodes* spp. cell lines could be due to insufficient sequence similarity between the tick genera, such that the primers designed for *R. microplus* did not bind to the gene in DNA or RNA extracted from these cell lines. A search for adrenergic octopamine receptor genes in *Ixodes scapularis* (https://www.vectorbase.org/organisms/ixodes-scapularis and http://www.ncbi.nlm.nih.gov/nuccore) found nothing, but there were numerous putative G-protein coupled octopamine receptors. Alternatively, the *βAOR* gene may be absent from these species or genera. It is more likely that the annotation for β-adrenergic-like octopamine receptor is incomplete.

The *R. microplus* cell lines have proven to be useful tools for investigating tick genetics in relation to acaricide resistance as evidenced from the findings of this study and other reports [18, 21, 22]. This study has also shown that cultures established from field populations have the potential to include and fix different variants of the same genes, such that some care is needed in interpretation of the results.

## Conclusions

The present study has identified a new βAOR gene and demonstrated a higher ABCB10 expression level in the BME/CTVM6 cell line, indicating that tick cell lines provide a useful experimental tool for acaricide resistance studies and further elucidation of tick genetics.

## Abbreviations

aa, amino acid; ABCB10, ATP-binding cassette B10; ANOVA, analysis of variance; bp, base-pair; cDNA, complementary deoxyribonucleic acid; gDNA, genomic deoxyribonucleic acid; NTC, non-template control; PCR, polymerase chain reaction; qPCR, real-time quantitative PCR; βAOR, β-adrenergic octopamine receptor; SNP, single nucleotide polymorphism

## Additional files

Additional file 1: Figure S1.
*βAOR* gene in earlier passages of *Rhipicephalus* cell lines BME/CTVM5 and BME/CTVM6. Detection of *βAOR* gene in *Rhipicephalus microplus* cell lines BME/CTVM5 passage 8 (5p8) and BME/CTVM6 passages 32, 221 and 243 (6p32, 6p221 and 6p243, respectively). Amplicons of 183 bp, 220 bp and 245 bp were detected in the gDNA of BME/CTVM5 and amplicon of only 220 bp was detected in BME/CTVM6 passages. M = Marker. (TIF 2846 kb) Additional file 2: Figure S2.
*βAOR* sequences of the 245 bp amplicon from BME/CTVM5 genomic DNA. There were two regions of heterogeneity from the sequencing traces of the alternative 245 bp amplicon detected in BME/CTVM5, indicating the existence of multiple primer binding sites for the sequencing reactions. (TIF 9962 kb)
