# Supplementary figures and images for: Identification of a novel β-adrenergic octopamine receptor-like gene (βAOR-like) and increased ATP-binding cassette B10 (ABCB10) expression in a Rhipicephalus microplus cell line derived from acaricide-resistant ticks
Source: Parasit Vectors. 2016 Aug 2;9:425. doi: 10.1186/s13071-016-1708-x (PMC4970269; doi:10.1186/s13071-016-1708-x)

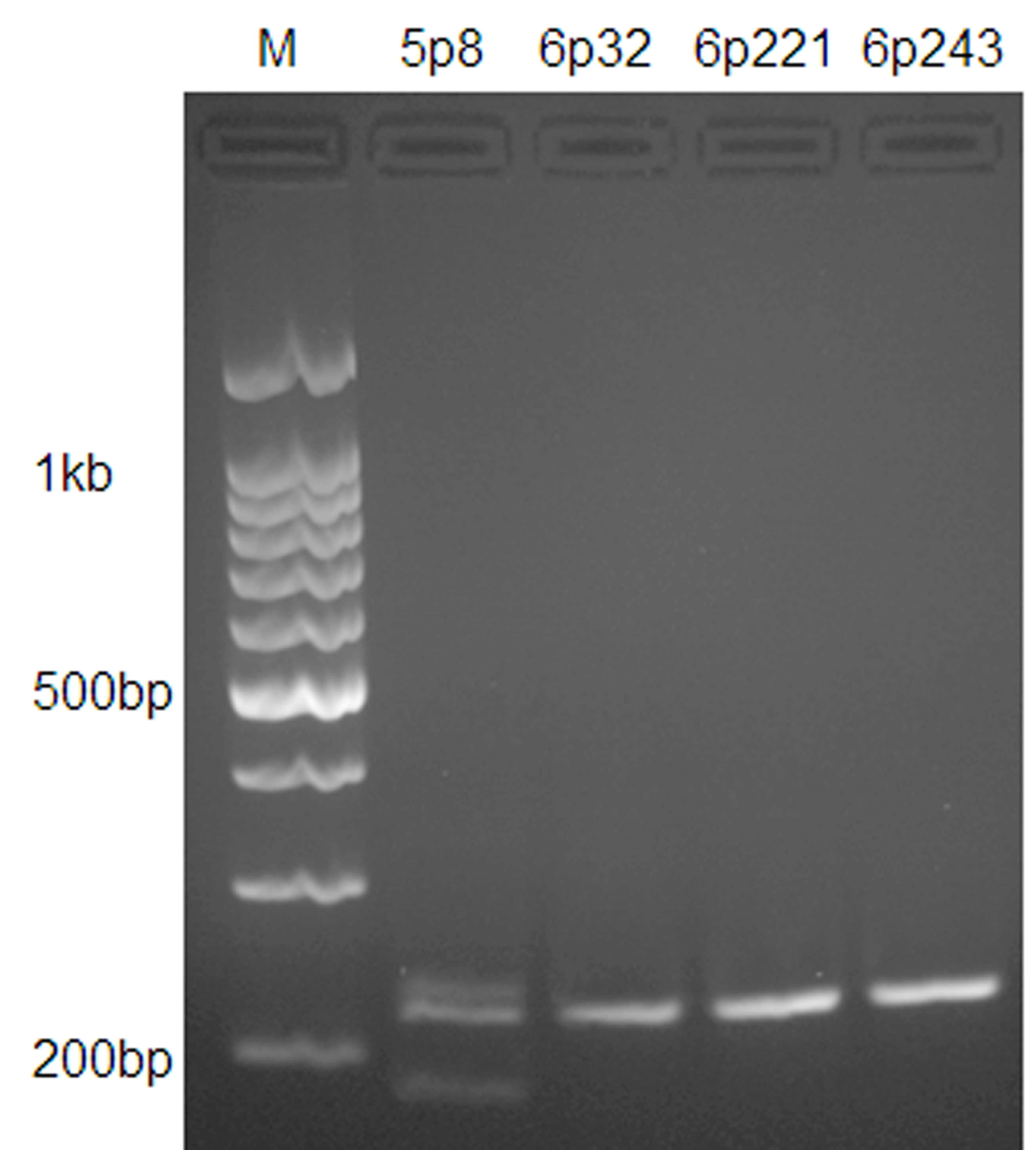

Supplement: Additional file 1: Figure S1. — βAOR gene in earlier passages of Rhipicephalus cell lines BME/CTVM5 and BME/CTVM6. Detection of βAOR gene in Rhipicephalus microplus cell lines BME/CTVM5 passage 8 (5p8) and BME/CTVM6 passages 32, 221 and 243 (6p32, 6p221 and 6p243, respectively). Amplicons of 183 bp, 220 bp and 245 bp were detected in the gDNA of BME/CTVM5 and amplicon of only 220 bp was detected in BME/CTVM6 passages. M = Marker. (TIF 2846 kb) [file 13071_2016_1708_MOESM1_ESM.tif]

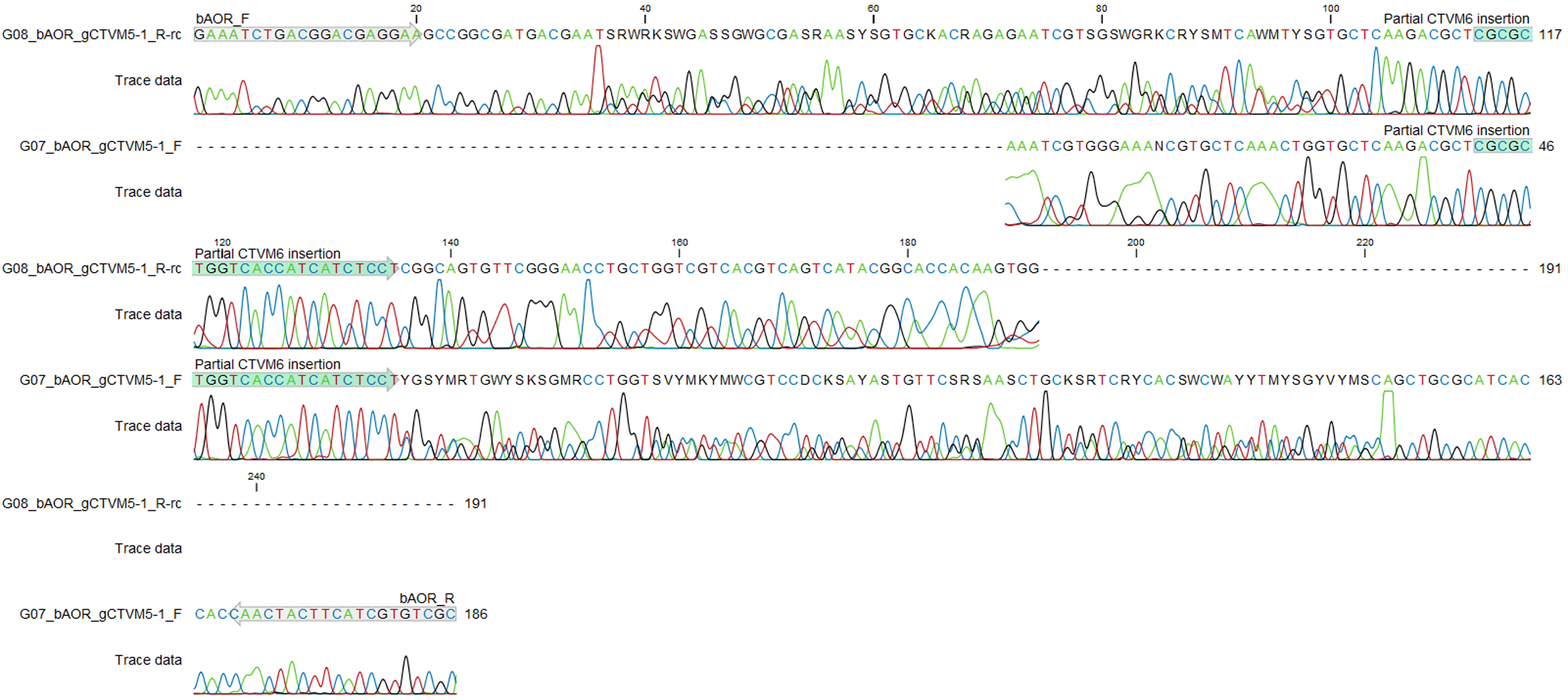

Supplement: Additional file 2: Figure S2. — βAOR sequences of the 245 bp amplicon from BME/CTVM5 genomic DNA. There were two regions of heterogeneity from the sequencing traces of the alternative 245 bp amplicon detected in BME/CTVM5, indicating the existence of multiple primer binding sites for the sequencing reactions. (TIF 9962 kb) [file 13071_2016_1708_MOESM2_ESM.tif]
